# Digestion

**Published:** 1866-03

**Authors:** J. P. Holmes

**Affiliations:** Jackson, Miss.


### DIGESTION.

BY DR. J. P. HOLMES, JACKSON, MISS.

DIGESTION is the process by which the food we take for our nourishment is brought into that condition in which it may be taken up or absorbed from the alimentary canal by the vessels specially appointed for that function. Unlike the process in the vegetable kingdom; which require, for their nutrition, only a constant supply of inorganic substances, such as water, carbonic acid, saline matters, and the like, which, being already prepared for absorption, are taken in by the plants, and readily converted into material, suitable for their maintenance and growth, requiring no preliminary modification or complex physical structure. In man, for whom simple inorganic substances alone will not furnish a support, but who requires organic materials which have previously formed a part of animal or vegetable bodies, this, too, in a solid form, and which, before preparation, has to undergo numerous modifications, as a consequence, there is required a more complex alimentary arrangement for proper digestion. Formed and brought into existence by One whose attributes are Omnipotence, Omnipresence and Omniscience, the physical structure of man, is deficient in no one respect, but has provided functions and relations necessary to meet every exigency — the study of which cannot fail to impress one with wonder at the display of wisdom in his Creator. His anatomical structure has provided all the functions for locomotion and labor to facilitate his efforts for support; and in the alimentary process there is wanting nothing to reduce his food to the condition for nourishment, In his mouth, the first receptaculum pabuli, he has provided a set of teeth, by which, in their mechanical structure, he can readily masticate his food, which, after maceration and insalivation, passes down through the fauces into the pharynx and œsophagus into the stomach. The stomach, which is said to be an expansion of the œsophagus, is provided with such a fibrous structure that the food being brought into it undergoes a continual rotation, so that every particle is impregnated with gastric juice, the second fluid in the operation of digestion. From the stomach the remainder of the mass, in a state of chyme, a part having undergone the assimilative modification for nutrition and absorbed by appropriate vessels, passes on down first into the duodenum, where it meets with the pancreatic juice and the bile. There it undergoes such modifications that the chyle, which is the nutritive element, is absorbed by the chyliferous vessels, which arise at the mucous surface of the intestines, and is passed through the mesenteric glands to the thoracic duct, and finally passes into the left subclavian, thence on through other vessels, it undergoes purification and other modifications to its office of nutrition. The remaining chyme, passing on by the peristaltic motion of the bowels, meets with the intestinal juice, the fifth and last fluid in digestion, and undergoes other modifications and extractions. From the duodenum it passes through the jejunem, ileum, cæcum, colon and rectum, finally, as excrementitious matter out through the anus. A consideration of these functions readily substantiates the conclusion that man was intended for a long and happy age; and were it not on account of his base irregularities and inattentions, the average age of man, instead of being the short thirty-three years, would easily be lengthened to the primitive three score and ten. Disease mainly originates in improper digestion, and the first loose cog is found in the hasty and improper modification of the food, which is more easily liquified when reduced to small particles, for then it can be brought in contact, in every part, with the fluids of the mouth and stomach; but if this is not done, it passes into the stomach in a solid form—there remaining an incubus originating impure gases, and causing a disarrangement of the whole system. These gasses or acids seem not only to vitiate the circulation, a proper condition of which is the *sine qua non* of health; but they also, by a reflex action, are brought into contact with the teeth, and it is by means of these chemical agents that caries and the numberless other diseases to which the tooth is heir, are superinduced. It is evident, then, that good teeth and proper mastication are the Palladium of a sound body and a sound mind (*sana mens in sana corpora*), for the latter is a dependent on the former. Of this fact, no people seem to be so forgetful as the American. They seem to act on the principle of eating to live, and the act itself is more a task than a pleasure. Seated at the dining table, haste and silence is the rule of action. They gulp down mouthful after mouthful, and forcing it down into the stomach, forgetful of hygienic principles, crowd it to such an extent and excess that its energies, being overtaxed, it does not perform its functions, and digestion is but half accomplished. Resulting from this, we every day meet pale and cadaverous individuals, whose anæmic diathesis too clearly indicates the embodiment of disease, their trembling limbs scarcely able to support their emaciated frame ; their colorless lips, and the absence of the bright sparkling of the eye, all evidence a gross inattention or ignorance of the primary principles of health. A remedy of these evils devolves not less on the dentist than the physician. The importance, however, of this profession, until late years, has been totally ignored, and the prerogative of benefiting the human race was claimed by the medical profession; but the earnest and successful efforts of the bright luminaries in the dental profession have raised her standard and importance to a level with her sister. If, and it is to be hoped they will, the younger members who are about to assume the paraphernalia of graduation will, by diligent study and research, seek to elevate their alma mater still higher, the importance of the dental profession will continue to increase, and the primary, principles of health will be more extensively disseminated.

February, 1866. Cincinnati, Ohio.

